# Quantifying attention span across the lifespan

**DOI:** 10.3389/fcogn.2023.1207428

**Published:** 2023-06-22

**Authors:** Alexander J. Simon, Courtney L. Gallen, David A. Ziegler, Jyoti Mishra, Elysa J. Marco, Joaquin A. Anguera, Adam Gazzaley

**Affiliations:** 1 Neuroscape Center, University of California, San Francisco, San Francisco, CA, United States; 2 Department of Neurology, University of California, San Francisco, San Francisco, CA, United States; 3 Weill Institute for Neurosciences & Kavli Institute for Fundamental Neuroscience, University of California, San Francisco, San Francisco, CA, United States; 4 Department of Psychiatry, University of California, San Diego, San Diego, CA, United States; 5 Department of Neurodevelopmental Medicine, Cortica Healthcare, San Rafael, CA, United States; 6 Department of Radiology, University of California, San Francisco, San Francisco, CA, United States; 7 Department of Psychiatry, University of California, San Francisco, San Francisco, CA, United States; 8 Department of Physiology, University of California, San Francisco, San Francisco, CA, United States

**Keywords:** sustained attention, vigilance decrement, attention span, continuous performance task (CPT), attentional modeling

## Abstract

**Introduction:**

Studies examining sustained attention abilities typically utilize metrics that quantify performance on vigilance tasks, such as response time and response time variability. However, approaches that assess the duration that an individual can maintain their attention over time are lacking.

**Methods:**

Here we developed an objective attention span metric that quantified the maximum amount of time that a participant continuously maintained an optimal “in the zone” sustained attention state while performing a continuous performance task.

**Results:**

In a population of 262 individuals aged 7–85, we showed that attention span was longer in young adults than in children and older adults. Furthermore, declines in attention span over time during task engagement were related to clinical symptoms of inattention in children.

**Discussion:**

These results suggest that quantifying attention span is a unique and meaningful method of assessing sustained attention across the lifespan and in populations with inattention symptoms.

## 1. Introduction

The ability to maintain a stable state of attention while performing a mundane activity is often referred to as sustained attention (SA) or vigilance (Mackworth, 1948; Langner and Eickhoff, 2013; Esterman et al., 2014). SA plays a crucial role on performance in real-world situations, such as driving, academic settings, and success in the workplace (Wei et al., 2012; Divekar et al., 2013; Clayton et al., 2015). Objective metrics that quantify different aspects of SA may provide useful information for how individuals engage in daily activities (e.g., conduct on our roads, school curriculum, and workplace policy) with cognitive limitations in mind. For instance, receiving feedback about when SA wanes can help signal when a break may be beneficial.

Studies that have examined SA have historically focused on response time (RT) metrics, such as average RT and response time variability (RTV), while participants perform vigilance tasks that require continuous attention (McAvinue et al., 2012; Staub et al., 2013; Fortenbaugh et al., 2015). In addition to traditionally used RT based metrics, measures derived from signal detection theory, such as D’, are commonly used to assess performance accuracy during sustained attention tasks (Fortenbaugh et al., 2015). While these metrics inform us about an individual’s *overall* performance during a SA task, they do not provide information about how long one can maintain their attention *over time*. Some studies have assessed how performance in the RT metrics change over the course of a SA task by quantifying “vigilance decrements” (Parasuraman et al., 1989; Tucha et al., 2009; Langner and Eickhoff, 2013; Rosenberg et al., 2013; Wang et al., 2014). These studies have demonstrated that performance on SA tasks decline over time (Mackworth, 1948), that this worsening in task performance over time reflects cognitive fatigue (Wang et al., 2014), and that it may be exacerbated by conditions that affect attention, such as normal aging and ADHD (Parasuraman et al., 1989; Huang-Pollock et al., 2012; Langner and Eickhoff, 2013). While insightful, these types of analyses still do not quantify the amount of time that an individual is able to maintain a stable optimal attentional state, and thus do not yield a direct, objective metric of attention span (A-span)—the length of time that an individual can maintain an optimal attentional state.

Although the phrase “attention span” is commonly used by the general population to describe the ability to sustain attention, methods to objectively quantify this capacity in both research and clinical settings are largely lacking. To this end, we defined a new metric to quantify an individual’s attention span (A-span): how long one is able maintain a state of optimal attention, defined as a period of high performance without response errors and consistent RTs. We specifically calculated an individual’s A-span by assessing the maximum length of time that a participant was able to maintain this optimal attentional state while performing a visual continuous performance task (CPT), a commonly used vigilance task in which participants respond to frequently occurring targets and withhold responses to infrequent non-targets (Esterman et al., 2013, 2014). We also quantified vigilance decrements in A-span to examine changes in A-span over the course of the CPT (“A-span decrements”).

Here, we leveraged a large dataset from children, young adults, and older adults to examine how A-span captures attention abilities. First, we compared A-span to traditional metrics of SA performance (i.e., RT and RTV) in a population of young adults. We then tested the hypothesis that A-span measures would follow an inverted-U pattern across the lifespan, such that it peaks in young adulthood and is reduced in older adults and children. Changing in a similar manner as traditional metrics would suggest that A-span metrics are sensitive to detecting age-related SA changes (McAvinue et al., 2012; Staub et al., 2013; Fortenbaugh et al., 2015). Finally, we evaluated the clinical utility of these metrics by examining if there were relationships between A-span measures and real-world symptoms of inattention in children, as indexed by the Vanderbilt ADHD Diagnostic Rating Scale (VADRS), given that SA impairments are well documented in individuals with ADHD (Huang-Pollock et al., 2006, 2012). In doing so, we assess whether A-span can serve as a unique and meaningful approach to evaluate SA abilities in separate age groups across the lifespan and in populations with attention impairments.

## 2. Materials and methods

### 2.1. Participants

We compiled CPT data from a series of studies recently performed at the UCSF Neuroscape Center by the present authors, with a total of 68 children (mean age = 9.57 +/− SD 1.62 years, range 7–13 years; 15 female, 53 male) recruited from 3 studies (Gallen et al., 2021; Mishra et al., 2021; Anguera et al., 2023), 88 young adults (mean age = 25.02 +/− SD 2.96 years, range = 19–32 years; 55 female, 33 male) recruited from 3 studies (2 of which have been published Ziegler et al., 2019; Mishra et al., 2021), and 106 older adults (mean age = 68.49 +/− SD 6.45 years, range = 56–85 years; 50 female, 56 male) recruited from 2 studies (1 of which has been published Anguera et al., 2022). See Supplementary material for more information about the studies in which the CPT data reported here were collected.

All participants had normal or corrected-to-normal vision, had no history of stroke, traumatic brain injury, or psychiatric illness (except for diagnosed ADHD), and were not taking psychotropic medication, except for 8 children who were taking stable doses of ADHD medication during their participation in the study. Additionally, older adult participants were screened for severe cognitive impairment using a Montreal Cognitive Assessment (MOCA) cutoff score of 18 (Trzepacz et al., 2015) and a composite score from a battery of neuropsychological tests (see Supplementary material for more information). All participants and their parents and/or legal guardians (for all children under the age of 16) gave informed consent to participate in the study according to procedures approved by the Committee for Human Research at the University of California San Francisco. The methods employed in this study were performed in accordance with the relevant guidelines specified in the Declaration of Helsinki.

### 2.2. Paradigm and stimuli

**FIGURE 1:**
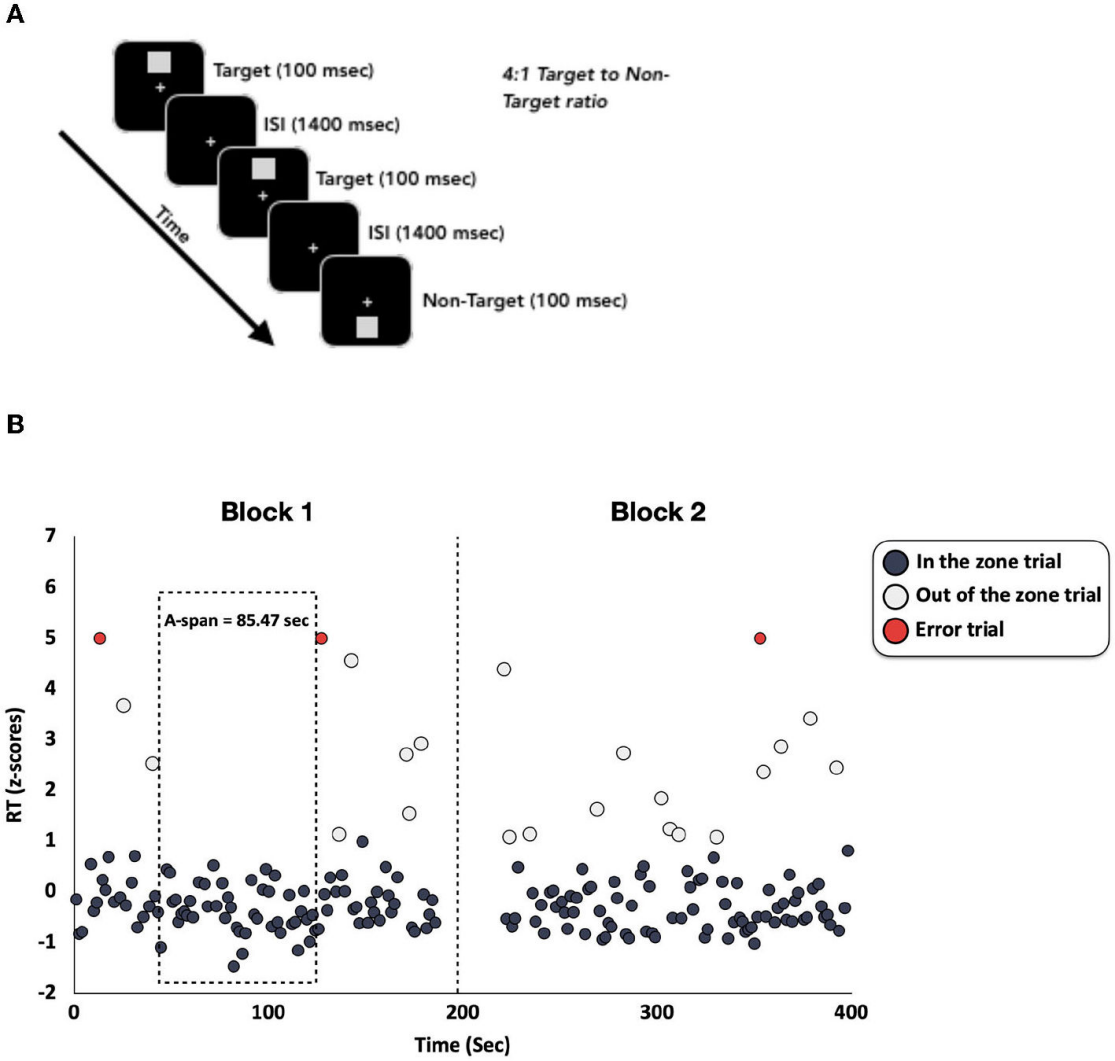
**(A)** Stimuli and protocol for the CPT. There were a total of 250 trials, with 80% targets and 20% randomly occurring non-targets. **(B)** Z-scored RTs from an example participant. Each RT was z-scored and plotted over time. RTs that are faster than 1 z-score above the mean are plotted in dark gray and are labeled as “in the zone” trials. RTs slower than 1 z-score above the mean are plotted in light gray and are labeled as “out of the zone” trials. Trials in which there was an error were plotted in red and were labeled as “error trials”. The dashed vertical line represents the break between the first and second CPT blocks. The dotted box highlights the longest period during the CPT when this participant was able to maintain an “in the zone” state (i.e., their A-span).

Participants from all age groups completed the same visual CPT in the same research lab at the UCSF Neuroscape Center (Figure 1A), except for 16 children who completed the same CPT using identical equipment at Cortica Healthcare’s labs in Marin County. The CPT was modeled after the Test of Variables of Attention (TOVA) (Leark et al., 2007) and has been used in several previously published studies from our group (Anguera et al., 2013, 2017a,b; Ziegler et al., 2019). The CPT was programmed in Presentation (http://neurobs.com) and the stimuli consisted of light gray squares that appeared on a black background at either the top or bottom half of the computer screen (see Figure 1A). Participants were instructed to respond to target stimuli (squares at the top half of the screen) with the spacebar and to withhold responses to non-target stimuli (squares at the bottom half of the screen). Each stimulus remained on the screen for 100 milliseconds, with a 1,400 millisecond inter-trial-interval. The CPT consisted of two conditions: The first condition had infrequent target stimuli (a 1:4 target to non-target ratio), while the second condition had frequent target stimuli (a 4:1 target to non-target ratio). For our analyses here, we only analyzed the condition with frequent targets to maximize the number of trials with correct (target) RT values, which are required for a precise A-span measurement. In this CPT condition, participants completed 2 blocks that each contained 125 total trials (100 targets and 25 non-targets) per block. The blocks were separated by a brief break in the task. The break was included to maintain consistency with the TOVA. Across the entire CPT condition, there were a total of 200 targets and 50 non-targets and took 6 min and 15 seconds to complete.

### 2.3. Computing traditional attention metrics

We computed traditional SA metrics, average RT and RTV (the standard deviation of RTs), for all correct responses to target stimuli across the entire CPT. RTs that were faster than 150 msec were excluded from the traditional metric computations, as this is often considered too fast for accurate perceptual discrimination and thus likely reflects a more error-prone state (Leark et al., 2007). We also computed RT and RTV in each of the 2 blocks separately to examine vigilance decrements (defined as the percent change in RT and RTV from the first to the second block).

### 2.4. Computing A-span

We computed the novel A-span metric using custom MATLAB code that built upon an approach commonly used in the literature to quantify moment-to-moment fluctuations of attention (Esterman et al., 2013, 2014; Kucyi et al., 2017). This approach characterizes when a participant is “in the zone” or “out of the zone” (defined below) using trial wise accuracy and RT (Figure 1B). Here, we extended this approach to characterize an individual’s A-span by computing the maximum amount of time that a participant was able to maintain an “in the zone” state without deviating to an “out of the zone” state.

To quantify A-span, we first *z*-scored the correct RTs at the single participant level. Any correct RT that fluctuated around the average RT and was faster than 1 *z*-score above an individual’s average RT was characterized as an “in the zone” trial. RTs that were slower than 1 *z*-score were characterized as “out of the zone” trials. Trials when the participant made an error were characterized as “error trials”. RTs that were faster than 150 msec were also characterized as “error trials”, since this is considered to be too fast for accurate perceptual decision making (Leark et al., 2007). All “error trials” were categorized as contributing to the participant being not “in the zone”, as incorrect responses in CPTs reflect a drift of attention away from the task (Robertson et al., 1997; Smallwood and Schooler, 2006; Esterman et al., 2013). Additionally, if a stretch of “in the zone” trials was punctuated by the break between blocks, we considered that as the end of the “in the zone” segment because the absence of task demands during the break meant that they were no longer in an optimal task-engaged state. We next computed the maximum amount of time (in seconds) that a participant was able to maintain an “in the zone” optimal attentional state (spanning at least 2 consecutive trials). We refer to this duration of time throughout this manuscript as “A-span”. Though it was not examined in the present study, the average amount of time that a participant can stay “in the zone” (i.e., average A-span) may also be a meaningful approach of measuring A-span (see Supplementary material for more information). As with th traditional attention metrics, we computed these A-span metrics across the entire CPT. We also examined vigilance decrements in A-span (percent A-span change between the first and second task blocks). Additional details regarding the A-span calculations can be found in Supplementary material. We then examined whether this new metric was distinct from traditional SA metrics (e.g., RT and RTV). Further, we asked how these A-span metrics differed across age groups and how they were related to symptoms of inattention in children.

### 2.5. Characterizing inattention symptoms in children

For 44 of the 68 children, we also collected parent ratings of inattention in the real world using the Vanderbilt ADHD Diagnostic Rating Scale (VADRS-IA). ADHD symptoms were assessed using 18 questions that probed the frequency that the child displays various ADHD symptoms, with questions 1–9 assessing inattentive symptoms and questions 10–18 assessing hyperactive/impulsive symptoms. Parents rated each symptom on a scale of 0 (“Never”) to 3 (“Very Often”). Given our interest in SA, we focused on relating the inattentive symptoms (questions 1–9) to A-span performance metrics. Therefore, we correlated our A-span metrics with the number of positive responses (a 2 “Often” or 3 “Very Often”) on the 9 questions that probe inattention symptoms (Wolraich et al., 2003). Of the 8 children in this study who were taking ADHD medication at the time of data collection, only 1 of them provided VADRS-IA data. Therefore, we did not control for medication status during this analysis.

### 2.6. Statistical analysis

All statistical analyses were conducted in IBM’s SPSS Statistics 20 software. First, we examined A-span metrics within each age group independently. We assessed whether there were significant A-span decrements across the CPT (i.e., if the percent change scores significantly differed from 0) using Wilcoxon signed rank tests. We chose to use this non-parametric approach to reduce the influence from potential extreme values. Since the Wilcoxon signed rank test compares our sample median against a hypothetical median, we highlighted the median percent change scores when reporting A-span decrements in each age group.

We then evaluated relationships between traditional and A-span metrics by conducting Spearman correlations between these metrics in young adults only. We chose to use Spearman correlations to reduce the influence that potential extreme values had on the correlations (Akoglu, 2018). Additionally, Bayesian non-parametric correlations were conducted to test the independence between A-span and traditional metrics.

To examine age group differences on A-span and traditional metrics, we conducted one-way ANOVAs on each metric with a between-subjects factor of age group (children, young adults, and older adults). We followed these analyses with an interrogation of pairwise differences between age groups with independent samples *t*-tests (see Supplementary material).

Finally, to evaluate the clinical utility of A-span metrics in children, we examined the relationship between these metrics and clinically-used inattention symptoms, as indexed by the number of positive responses to the VADRS-IA that these children displayed, using Spearman correlations. To determine if the relationships between attention span metrics and inattention symptoms were stronger than the relationships between traditional metrics and inattention symptoms, we converted Spearman correlation coefficients to Pearson correlation coefficients (Myers and Sirois, 2004), and then formally compared the correlation coefficients (Pearson and Filon, 1898; Diedenhofen and Musch, 2015). For each set of analyses where we ran multiple statistical tests (e.g., correlations between inattentive symptoms and both A-span metrics), we corrected *p*-values using an FDR correction for multiple comparisons and used a two-tailed significance threshold of *p* < 0.05.

## 3. Results

### 3.1. Characterizing A-span across the lifespan

We began by calculating and characterizing the new A-span metrics in each age group separately (Table 1). We found that children had an A-span of 29.61 seconds, which declined significantly (−27.41%) over the course of the CPT (*Z* = 687.00, *p* = 0.003). Young adults had an A-span of 76.24 seconds, which did not decline significantly (−2.54%) over the course of the CPT (*Z* = 2,193.00, *p* = 0.328). Finally, the older adults had an A-span of 67.01 seconds, which also did not decline significantly (−8.40%) over the course of the CPT (*Z* = 2,672.00, *p* = 0.606). Although the median A-span percent change was negative in each of the age groups, there were several participants who experienced very large increases in A-span (>100%) throughout the CPT. Most of these participants were young adults (*n* = 15 out of 88), while fewer were older adults (*n* = 7 out of 106), and the fewest were children (*n* = 2 out of 68).

### 3.2. Determining the uniqueness of A-span and A-span decrements in young adults

**FIGURE 2:**
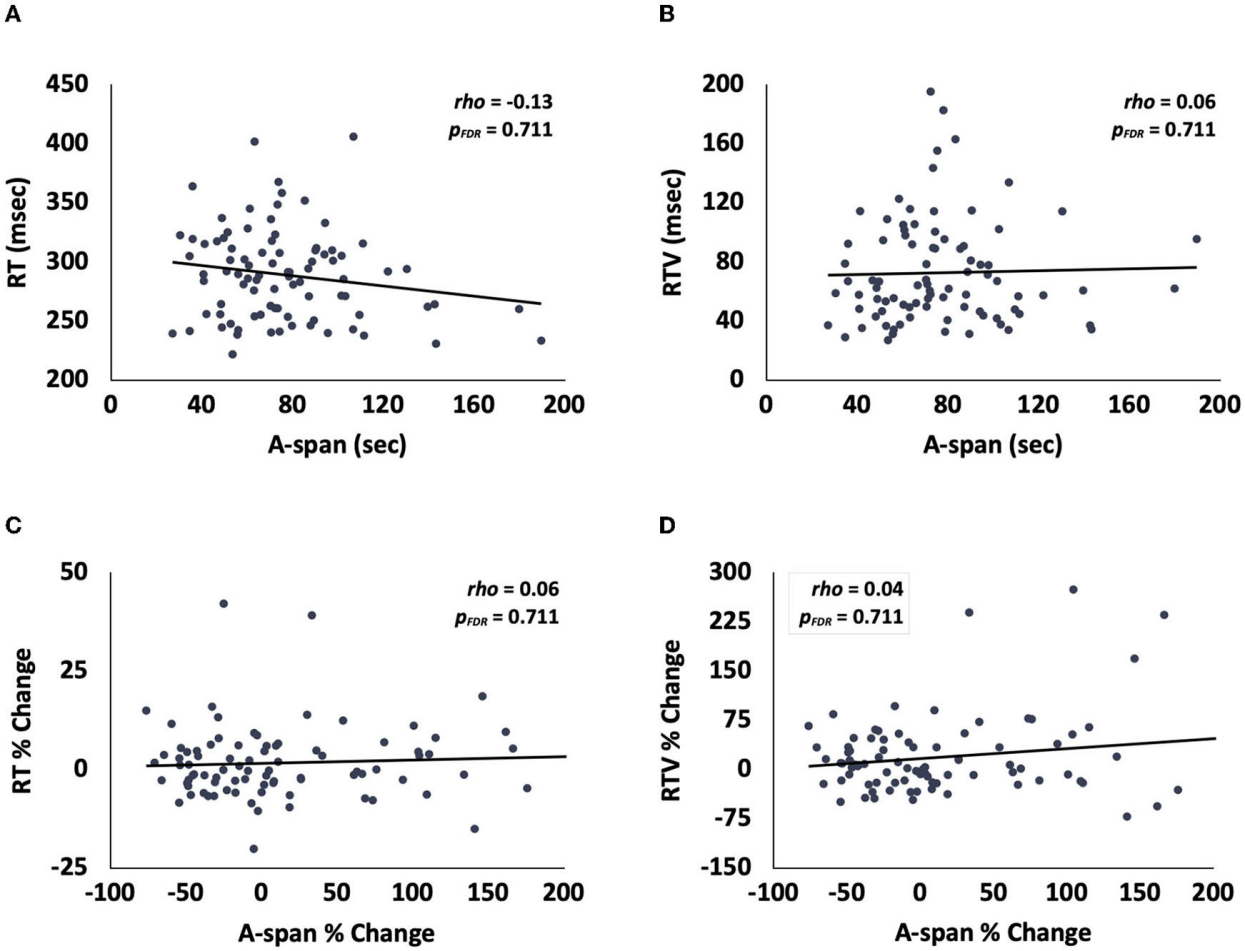
Scatterplots showing that, in young adults, **(A)** A-span was unrelated to RT and **(B)** RTV, and that A-span percent change was unrelated to **(C)** RT percent change and **(D)** RTV percent change.

We then assessed the relationships between A-span and traditional SA metrics in a population of young adults to determine the uniqueness of the new A-span metrics. We found that A-span was not correlated with RT or RTV [Figure 2A; RT: *rho*_(88)_ = −0.13, *p_FDR_* = 0.711, BF_01_ = 3.46; Figure 2B; RTV: *rho*_(88)_ = 0.06, *p_FDR_* = 0.711, BF_01_ = 6.39]. Similarly, A-span percent change was not correlated with either RT or RTV percent change [Figure 2C; RT percent change: *rho*_(88)_ = 0.06, *p_FDR_* = 0.711, BF_01_ = 6.32; Figure 2D; RTV percent change: *rho*_(88)_ = 0.04, *p_FDR_* = 0.711, BF_01_ = 6.96]. Together, these findings suggest that A-span and A-span decrement metrics may be distinct from traditional metrics and their vigilance decrements.

### 3.3. Age group effects on A-span metrics

We then examined changes in A-Span across the three age groups to assess whether A-span metrics follow similar patterns of SA change across the lifespan as reported elsewhere (McAvinue et al., 2012; Staub et al., 2013; Fortenbaugh et al., 2015). We specifically examined age group effects for all CPT metrics, as well as for vigilance decrements in each metric from the first to second block of the task.

#### 3.3.1. A-span

**FIGURE 3:**
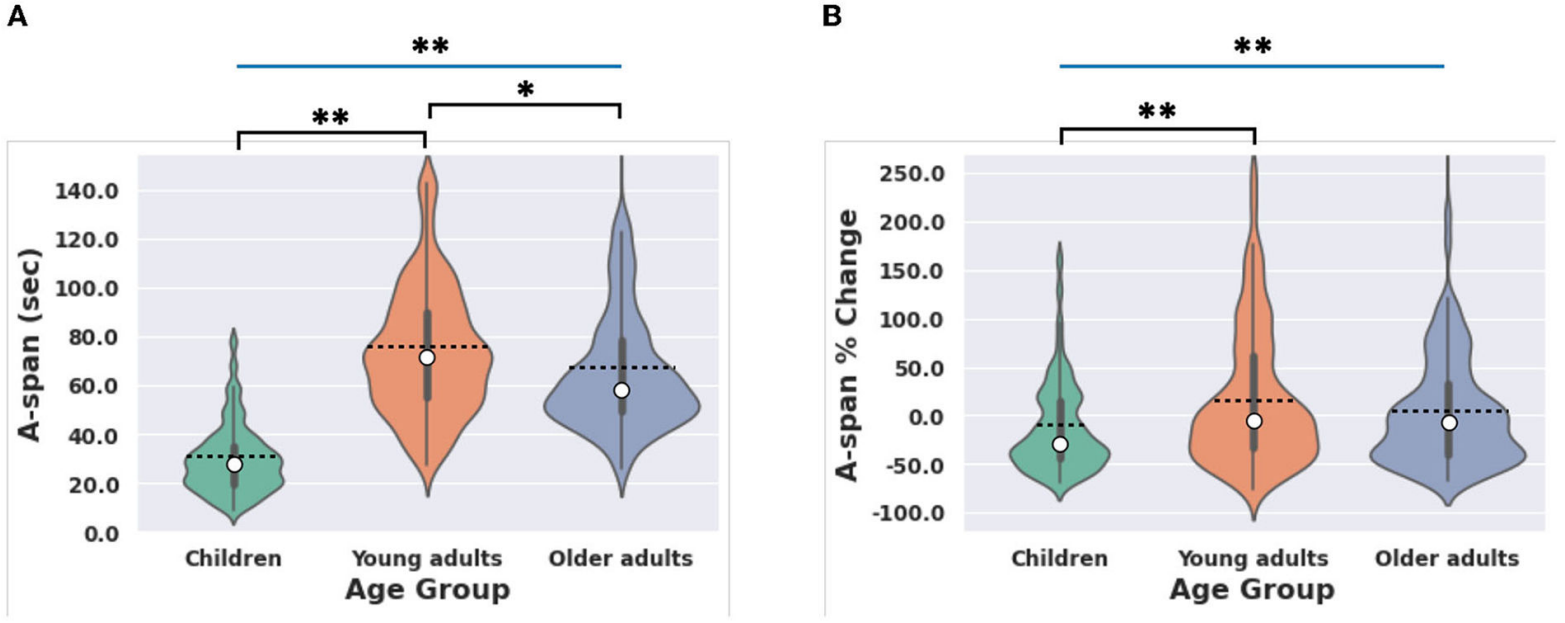
Age group effects on A-span metrics. **(A)** Age effects on A-span were driven by children and older adults having shorter A-spans than young adults. **(B)** Age effects on A-span percent change were driven by children having greater A-span decrements (i.e., a more negative A-span percent change) than young adults. Box and whisker plots represent the bounds of each quartile. Dashed lines represent the group average. White dots represent the group median. Blue significance bars indicate significant interactions revealed from the ANOVAs and black significance bars indicate significant *t*-test results. *p < 0.05, **p < 0.01.

First, we examined whether there were age group differences in A-span. A one-way ANOVA revealed a significant age group effect for A-span [Figure 3A; *F*_(2,259)_ = 66.32, *p* < 0.001, η^2^ = 0.34], such that young adults had longer A-spans than children and older adults. See Table 2 for details on pairwise comparisons between age groups. The age group effect on A-span was nearly identical when excluding children who were taking ADHD medication at the time of data collection [*F*_(2,251)_ = 66.23, *p* < 0.001, η^2^ = 0.34]. Additionally, the age group effect on A-span was similar when using an ANCOVA that used a type III sum of squares to control for differences in sample size between age groups while also setting the study in which the data were originally collected as a covariate [F_(2,262)_ = 33.96, *p* < 0.001, η^2^ = 0.21].

#### 3.3.2. Traditional metrics

Next, we confirmed that the traditional metrics (RT and RTV) also showed this expected pattern of changes across the lifespan (McAvinue et al., 2012; Staub et al., 2013; Fortenbaugh et al., 2015). One-way ANOVAs with a between-subjects factor of age group (children, young adults, and older adults) showed that there was a significant age group effect for RT [Supplementary Figure 3a; *F*_(2,259)_ = 110.30, *p* < 0.001, η^2^ = 0.46] and RTV [Supplementary Figure 3b; *F*_(2,259)_ = 264.03, *p* < 0.001, η^2^ = 0.67]. Similar to A-span, young adults had lower RT and RTV than children and older adults. See Supplementary material for statistics on pairwise comparisons between age groups. The similarities between the way that A-span and traditional metrics differ across age groups suggest that they may reflect distinct attentional processes that similarly fluctuate during development and aging.

#### 3.3.3. Decrements in A-span

We then examined whether A-span decrements followed this pattern of age group differences. A one-way ANOVA revealed a significant age group effect for A-span decrements, as indexed by A-span percent change [Figure 3B; *F*_(2,259)_ = 4.91, *p* = 0.008, η^2^ = 0.04]. Young adults experienced smaller A-span decrements than children but had similar A-span decrements as older adults. See Table 2 for details on pairwise comparisons between age groups. The age group effect on A-span percent change was similar when excluding children who were taking ADHD medication at the time of data collection [*F*_(2,251)_ = 6.27, *p* = 0.002, η^2^ = 0.05]. Additionally, the age group effect on A-span percent change was similar when using an ANCOVA that used a type III sum of squares to control for differences in sample size between age groups while also setting the study in which the data were originally collected as a covariate [F_(2,262)_ = 3.79, p = 0.024, η^2^ = 0.03].

#### 3.3.4. Decrements in traditional metrics

Next, we confirmed that vigilance decrements over time in traditional metrics followed the pattern of expected changes across the lifespan as previously reported (Parasuraman et al., 1989; Langner and Eickhoff, 2013). One-way ANOVAs with a between-subjects factor of age group (children, young adults, and older adults) showed that there was a significant age group effect for RT percent change from first to second block of the task [Supplementary Figure 3c; *F*(2,259) = 9.38, *p* < 0.001, η^2^ = 0.07]. Young adults had smaller RT percent changes (i.e., more stable performance throughout the entire CPT) than children but had similar RT percent changes as older adults. Unexpectedly, however, there was no effect of age for RTV percent change [Supplementary Figure 3d; *F*_(2,259)_ = 1.37, *p* = 0.257, η^2^ = 0.01]. See Supplementary material for statistics on pairwise comparisons between age groups. Like the metrics computed across the entire task, the similarities between the way that decrements in A-span and traditional metrics differ across age groups suggest that they may reflect distinct attentional processes that similarly fluctuate during development and aging.

### 3.4. Relationship between inattention symptoms and A-span decrements in children

We then assessed the potential clinical utility of A-span measurements by examining whether A-span metrics were related to real-world symptoms of inattention in children. We subsequently followed these analyses by testing for similar relationships between traditional metrics and inattention symptoms, to determine if the children included here exhibit similar SA deficits as reported elsewhere (Huang-Pollock et al., 2006, 2012).

#### 3.4.1. A-span metrics

**FIGURE 4:**
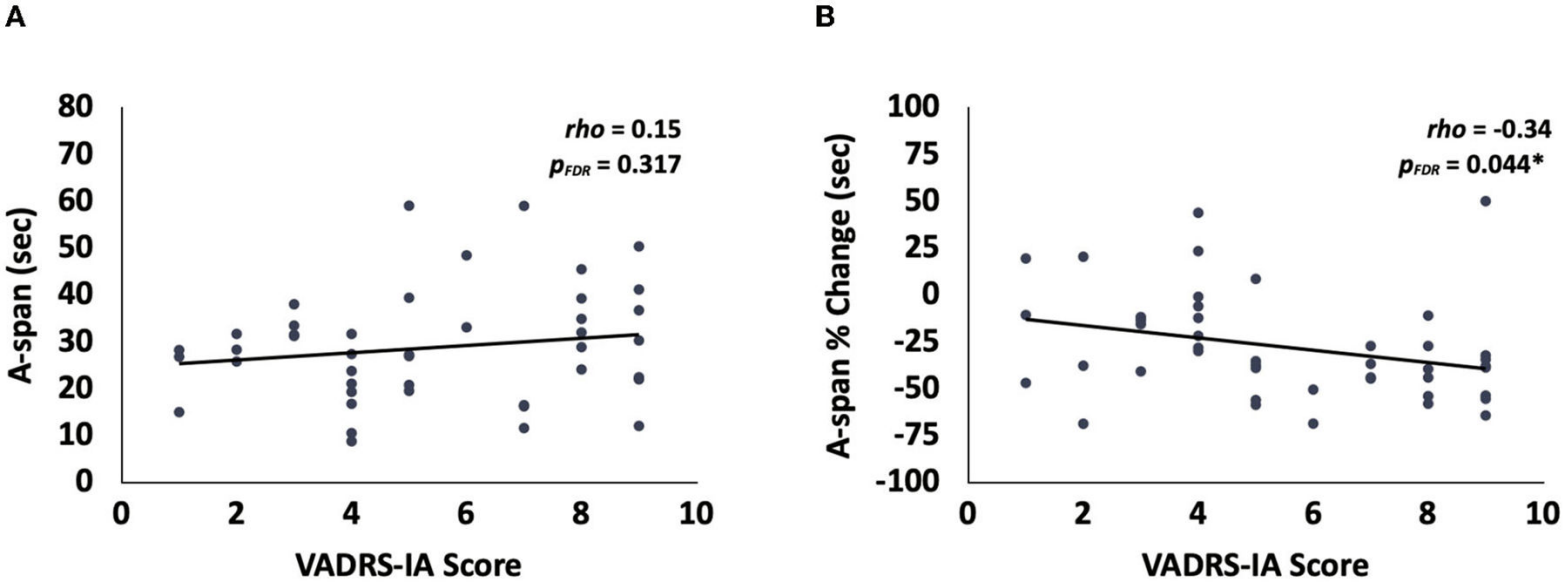
Relationships between A-span measures and inattention in children. **(A)** There was no significant relationship between the VADRS-IA score and A-span. **(B)** There was a significant relationship between the VADRS-IA score and the A-span % change. *p < 0.05.

We interrogated the relationships between each A-span metric and the number of inattention symptoms reported on the VADRS questionnaire. We found that the vigilance decrement in A-span was negatively related to ADHD-inattentive symptoms in children (i.e., a more negative A-span percent change was related to having more inattention symptoms) (Wolraich et al., 2003) [Figure 4B; *rho*_(44)_ = −0.34, *p_FDR_* = 0.044]. However, there was no relationship between A-span (i.e., across the entire task) and inattention symptoms [Figure 4A; *rho*_(44)_ = 0.15, *p_FDR_* = 0.317].

#### 3.4.2. Traditional metrics

Next, we sought to confirm that the traditional metrics showed similar relationships with inattention symptoms as documented elsewhere (McAvinue et al., 2012; Staub et al., 2013; Fortenbaugh et al., 2015). Interestingly, there was no relationship between any of the traditional metrics and inattention symptoms [Supplementary Figure 4a; RT: *rho*_(44)_ = 0.19, *p_FDR_* = 0.603; Supplementary Figure 4b; RTV: *rho*_(44)_ = 0.05, *p_FDR_* = 0.766; Supplementary Figure 4c; RT percent change: *rho*_(44)_ = 0.12, *p_FDR_* = 0.603; Supplementary Figure 4d; RTV percent change: *rho*_(44)_ = 0.15, *p_FDR_* = 0.603].

### 3.5. Inattention symptoms are more closely related to A-span percent change than traditional metrics

In an exploratory analysis, we sought to determine if the relationship between A-span percent change and inattention symptoms was significantly stronger than the relationships between traditional metrics and inattention symptoms. We found that the correlation between A-span percent change and inattention symptoms was significantly stronger than that for each of the traditional metrics and inattention symptoms (RT: *z* = −2.77, *p* = 0.006; RTV: *z* = −1.98, *p* = 0.047; RT % change: *z* = −2.11, *p* = 0.035; RTV % change: *z* = −2.43, *p* = 0.015).

## 4. Discussion

Here, we report a method of quantifying attention span by calculating the maximum amount of time that a participant was able to maintain an “in the zone” high performance state while performing a CPT. Our approach revealed that children had an A-span of 29.61 seconds, young adults had an A-span of 76.24 seconds, and older adults had an A-span of 67.01 seconds. Furthermore, A-span decrements were most pronounced in children, who experienced an A-span decline of −27.41% over the course of the CPT, while young and older adults experienced non-significant A-span decrements (−2.54 and −8.40%, respectively). A-span decrements were also sensitive to detecting inattention symptoms in children. The results we report here suggest that our approach of quantifying A-span is a unique and meaningful method of assessing SA abilities in separate age groups across the lifespan and in clinical populations.

### 4.1. A-span fluctuations across the lifespan

Although A-span performance followed previously seen patterns of change across the lifespan as the traditional metrics, A-span metrics were uncorrelated with traditional metrics in young adults. Bayesian analysis also provided evidence that A-span was independent from traditional metrics, suggesting that they may reflect distinct attentional processes. These findings are likely the result of two possible scenarios. First, A-span and traditional metrics may reflect different aspects of a common, more general, set of SA processes that change with development and aging. Second, these metrics may reflect distinct, unrelated cognitive processes that both happen to increase during development and decline during aging. Future work is warranted to address this question by identifying the neural activity profiles that facilitate A-span maintenance, as this type of interrogation would identify the similarities and differences between the neural correlates of A-span and traditional SA metrics, thereby enhancing our understanding of these cognitive processes.

Unexpectedly, we did not see any effects of age group on RTV vigilance decrements. Although many studies have shown that SA and vigilance decrements change across the lifespan (Parasuraman et al., 1989; McAvinue et al., 2012; Langner and Eickhoff, 2013; Staub et al., 2013; Fortenbaugh et al., 2015), there have been studies that have reported no SA changes with aging (Carriere et al., 2010). Thus, our results suggest that A-span might be more sensitive to detecting age-related vigilance decrements than RTV.

### 4.2. Clinical relevance of A-span

Importantly, we also observed that A-span percent change was related to inattentive symptoms in children, while traditional metrics were not. Further, the relationship with A-span percent change was significantly stronger than the correlations with traditional metrics. While declines in traditional metrics are well documented in individuals with ADHD (Huang-Pollock et al., 2006, 2012), null reports of SA deficits in ADHD populations do exist (Corkum and Siegel, 1993; Tucha et al., 2009). This inconsistency in the literature could be influenced by the heterogeneity of cognitive deficits in ADHD. Alternatively, traditional metrics may be too coarse to reveal group differences in a population with known elevated levels of performance variability (Huang-Pollock et al., 2012). It has been suggested that more granular approaches, such as vigilance decrements (Huang-Pollock et al., 2012), for assessing attention deficits in ADHD populations may be useful for better understanding how SA is impacted in ADHD. This new approach of A-span assessment may be a useful approach for assessing SA in ADHD given that it reflects how long an individual can hold their attention in an optimal state, and how this changes with time on task. However, these results should be interpreted with an abundance of caution. Future work should rigorously examine the reliability of using A-span measurements to detect inattention symptoms (Hedge et al., 2020).

Although we saw effects of age on A-span decrements, only children displayed significant A-span decrements over the course of the CPT (see Table 1). This finding highlights how children are poorer at maintaining stable attention over time relative to adults, and is even more intriguing when considering that A-span decrements in this age group are associated with symptoms of inattention. Together, these results suggest that A-span stability is sensitive to development, and impairments in an individual’s ability to maintain a stable A-span over time could be an important marker of attention impairments.

### 4.3. A-span as a new approach for assessing attention over time

Although traditional metrics that assess CPT performance are useful for detecting overall SA abilities, they do not directly quantify the ability to maintain uninterrupted attention over a sustained period of performance (Huang-Pollock et al., 2012). An individual’s average RT during a CPT could be fast because their psychomotor speed was fast while they were in an attentive state, but they could have had frequent lapses in attention that were not detected when computing an average RT across the whole CPT. Our finding that RT was uncorrelated with A-span in young adults supports this notion. Contrasting the neural correlates of A-span with what is known about the neural processes that underlie SA could further highlight how A-span differs from traditional metrics (Rosenberg et al., 2016; Helfrich et al., 2018). Many researchers have leveraged vigilance decrements to assess the extent of attentional decline over time (Parasuraman et al., 1989; Tucha et al., 2009; Langner and Eickhoff, 2013; Rosenberg et al., 2013; Wang et al., 2014). While this work has illuminated how performance in traditional metrics change over the course of a task, it has not helped researchers understand how the amount of time that an individual is able to maintain a stable optimal attentional state is relevant. Our new A-span metric achieves this while also providing an approach to quantify an ability that is seemingly intuitively understood amongst the general public.

When considering A-span as a measure of interest, researchers should consider the type of tasks that are aligned with its use. In general, CPTs, such as the SART, TOVA, and gradCPT (Leark et al., 2007; Carriere et al., 2010; Esterman et al., 2013, 2014), which have been used to assess metrics of SA, are likely to yield meaningful A-span measurements. These types of paradigms that sample a participant’s focus frequently (i.e., ones that require frequent responses) are more likely to capture brief fluctuations in attention, and thus will yield more precise A-span metrics. However, these tasks may index SA differently. Further research is necessary for determining which SA tasks are best suited for measuring A-span. Investigators should use caution when calculating A-span from more complex cognitive tasks (e.g., working memory, decision making, and interference resolution tasks). Longer RTs and errors in these types of tasks may not reflect attentional lapses, but instead may stem from other difficulties in cognitive processing, such as reaching working memory capacity limits or when there is uncertainty during complex decision making. Therefore, measuring A-span during a more challenging task might not purely reflect how long an individual can stay in an optimal SA state. Additionally, the task duration is an important factor to take into consideration when computing A-span. The CPT employed in this study was relatively short. A longer CPT may yield A-span measurements that reflect SA abilities differently. Utilizing CPTs that require less frequent responses may also provide meaningful, and potentially distinct, A-span calculations. However, since these types of CPTs have fewer trials, they will likely need to be longer than the task used in this study to obtain a precise A-span.

### 4.4. Future directions

Interrogating the similarities and differences in the neural processes underpinning A-span and traditional metrics is a potentially exciting future avenue of research. Several fMRI studies have implicated several widespread brain networks, including the default mode, salience, and dorsal attention networks, in maintaining “in the zone” attentional states (Esterman et al., 2013, 2014; Kucyi et al., 2017). Thus, these networks likely play a role in A-span maintenance. Additionally, incorporating recently developed neuroimaging analysis methods that are sensitive to detecting neural dysfunctions related to inattention into A-span studies can further illuminate how A-span is impacted by inattention (Cai et al., 2021). Ultimately, reaching a better understanding of how A-span decrements might be related to inattention could lead to better characterization of ADHD subtypes, and enhanced treatment personalization and efficacy (Leikauf et al., 2017; Griffiths et al., 2021).

Understanding how different task parameters contribute to A-span measurements is an important extension of this research. As described previously, future research should seek to identify whether longer tasks capture more meaningful A-span fluctuations than the A-span % change reported in this study. Establishing the minimum task length that can be used for calculating A-span is also an important avenue of future work. Finally, identifying the effects that taking a short break between blocks has on A-span decrements may illuminate how vigilance decrements may be mitigated or exacerbated.

### 4.5. Limitations

There are a few noteworthy limitations in this study. First, although we showed that a relatively short CPT (only 6 min and 15 sec in total) can yield meaningful A-span metrics, the optimal length of a CPT for measuring A-span (and decrements) remains to be determined. Computing A-span over longer periods in future work will allow us to understand more precisely how the rate and magnitude of A-span decrements might signify the presence of attention impairments. It is possible that some individuals who have short A-spans when measured on timescales of 5–10 min can maintain high task performance for several hours (or vice versa). Interestingly, some individuals experienced an increase in A-span with time on task. On the surface, this seems to contradict theoretical models of SA, such as the resources depletion theory (Esterman and Rothlein, 2019). A longer task might reveal that the amount of time it takes for an individual to reach their maximum A-span provides meaningful information regarding sustained attention abilities. Furthermore, it might reveal that the individuals who initially experienced large increases in A-span over time eventually show A-span decrements, thus capturing a “warm-up” period that has been reported in the SA literature (Kamza et al., 2019). It could also explain the disproportional distribution of these individuals across age groups that we observed here. Based on the present findings, future work examining individual differences in A-span dynamics over longer timescales is warranted to better understanding the utility of this metric in different scenarios. Ultimately, doing so could facilitate the use of A-span in real-world settings. Closed-loop systems can interpret shortening A-spans as an indication of a need to take a rest, or lengthening A-spans as a sign that an individual has yet to reach their maximum A-span.

Although we found evidence that A-span is unique from traditional measures, there are likely some individuals whose A-spans are affected by their RTV. For instance, an individual with frequent attentional lapses (i.e., slower responses) will likely have a shorter A-span than an individual with infrequent, but large lapses (i.e., several consecutive very slow responses), even though they may have similar RTV values. Understanding how the temporal distribution of variable responses impacts A-span measurements is a topic that future studies should examine more thoroughly. Moreover, the result that A-span is independent from traditional metrics should be interpreted with caution and replicated before concluding that A-span is truly measuring a unique aspect of SA that is not captured by traditional metrics.

Additionally, although we analyzed data from participants from a wide age range, we did not have any participants between the ages of 14–18 and 33–55. Therefore, it remains unknown how A-span and A-span decrements change during adolescence and middle adulthood. Finally, the present study did not examine the relative contribution of state (i.e., mood, fatigue, and stress) to A-span measurements. Future studies should seek to disentangle state vs. trait impacts on A-span.

## 5. Conclusion

Here, we demonstrated that A-span is a unique and meaningful index of SA abilities that differs between age groups across the lifespan, and that A-span decrements are related to clinical inattention symptoms in children. Our work suggests that A-span is a promising new approach for characterizing SA performance at the behavioral level, and should be further utilized when examining the effects of development and aging on SA abilities, and in clinical conditions that impact cognition.

## Supplementary Material

Supplementary Material

## Figures and Tables

**TABLE 1:** Descriptive statistics of A-span and A-span percent change for each age group.

		Children	Young adults	Older adults
A-span	Mean	29.61 sec	76.24 sec	67.01 sec
	Median	27.37 sec	72.17 sec	59.31 sec
	Stdev	13.86 sec	30.55 sec	28.28 sec
	Range	8.88–77.92 sec	27.12–189.74 sec	25.95–186.98 sec
A-span % change	Mean	−12.55%	20.02%	4.88%
	Median	**−27.41%**	**−2.54%**	**−8.40%**
	Stdev	46.73%	78.09%	61.62%
	Range	−68.45–160.25%	−76.21–346.08%	−67.20–299.38%
	*p*-value	0.003**	0.328	0.606

The row indicating “*p* value” reflects results from the Wilcoxon signed rank tests assessing if A-span percent change significantly differed from 0. **p < 0.01.

**TABLE 2:** Pairwise comparisons of A-span measures comparing young adults to children and older adults separately.

		Young adults vs. children	Young adults vs. older adults
A-span	*t*-statistic	*t*_(127.77)_ = −12.72	*t*_(192)_ = 2.18
	Cohen’s *d*	*d* = −1.89	*d* = 0.32
	*p*-value	*p* < 0.001**	*p* < 0.030*
A-span % Change	*t*-statistic	*t*_(145.69)_ = −3.23	*t*_(163.92)_ = 1.48
	Cohen’s *d*	*d* = −0.49	*d* = 0.22
	*p*-value	*p* = 0.002**	*p* = 0.142

* *p* < 0.05

** *p* < 0.01.

## Data Availability

The data analyzed in this study is subject to the following licenses/restrictions: The data were compiled from a series of recent studies conducted by the present authors. The data used to generate A-span measurements reported in this paper is available from the corresponding authors upon reasonable request. Requests to access these datasets should be directed to adam.gazzaley@ucsf.edu.
