# Supplementary material for: Quantifying attention span across the lifespan
Source: Front Cognit. Author manuscript; Available in PMC 2023 Nov 2. (PMC10621754; doi:10.3389/fcogn.2023.1207428)
Supplement: Supplementary Material [file NIHMS1938285-supplement-Supplementary_Material.docx]

**Supplemental Methods**

**Participants**

In this study, we pooled data from a CPT that has been commonly used as an outcome measure in several cognitive intervention studies in children, young adults, and older adults. The data that we analyzed in children, young adults, and older adults came from pre intervention assessments conducted by Neuroscape, Akili interactive, and Cortica Healthcare between 2015-2020 (4 of which have been published [1-4]. ﻿﻿

**Older adult eligibility screening**

In addition to the MOCA, older adults in this study completed a battery of neuropsychological assessments that tested psychomotor processing speed, task switching, immediate and delayed memory recall, and verbal fluency. Participants were excluded from study enrollment if they scored below 2 z-scores under the mean on any assessment, if they scored below 1.5 z-scores under the mean on any 2 assessments, or if they scored under an 18 on the MOCA.

**Characterizing faster trials as “in the zone”**

Contrary to the method used by in previous studies characterizing “in the zone” vs “out of the zone” trials [5], we categorized trials that were faster than 1 z-score below the mean RT also as “in the zone” instead of “out of the zone”, as long as they were not faster than 150 msec (which is considered to be too fast for accurate perceptual decision making [6]). We chose to modify this aspect of “in the zone” and “out of the zone” criteria because stimuli in the CPT used here appeared instantaneously and because participants here were instructed to respond as quickly as possible without sacrificing accuracy. In the studies that our analyses were based off of, stimuli would appear and disappear from the screen gradually [5, 7]. Thus, optimal RTs were more likely to occur closer to stimulus onset, which was evidenced by the existence of a rightward skew in RTs in all three age groups (**Supplemental figure 1**). Labeling these faster RTs as “out of the zone” would likely have resulted in many of them being miscategorized.

**
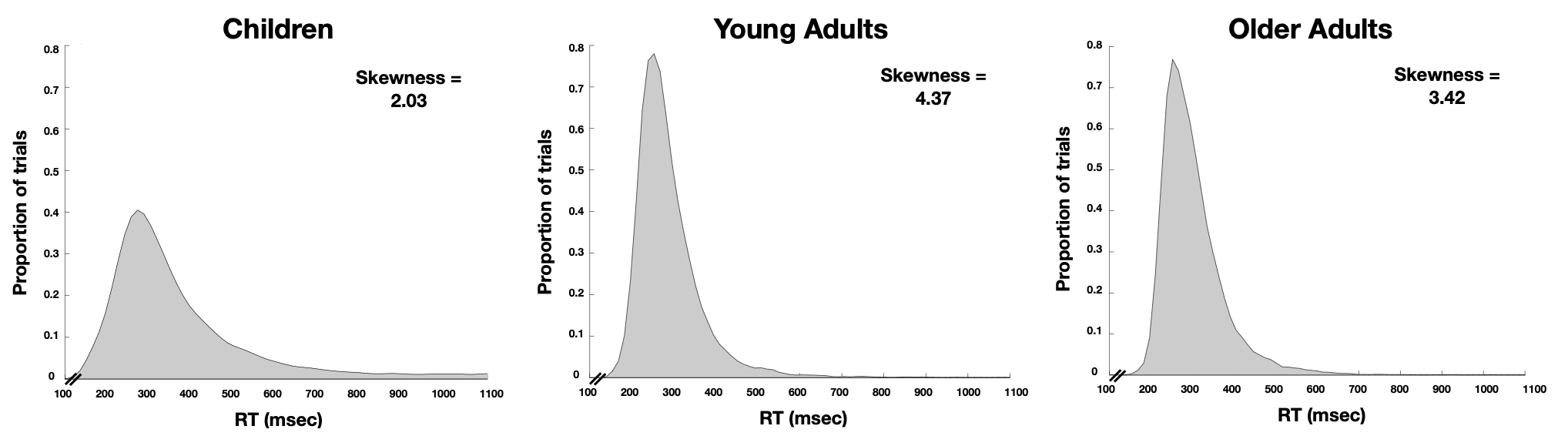
**

**Supplemental figure 1.** RT distributions from all three age groups during CPT performance. The rightward skew indicates that “out of the zone” trials were more likely to occur on trials with slow, as opposed to fast, RTs.

**Supplemental Results**

**Relationships between ‘maximum’ and ‘average’ A-span**

In addition to the maximum amount of time that an individual was able to maintain an “in-the-zone” state of optimal performance (i.e., maximum A-span), we also calculated the average amount of time of each of these “in-the-zone” stretches (i.e., average A-span). Maximum A-span was strongly correlated with average A-span in young adults (**Supplemental figure 2a**; *rho(88)* = 0.618, *p* < 0.001), as were the percent changes for each of these metrics (**Supplemental figure 2b**; *rho(88)* = 0.740, *p* < 0.001). This suggests that average A-span may also be a meaningful method for examining A-span.


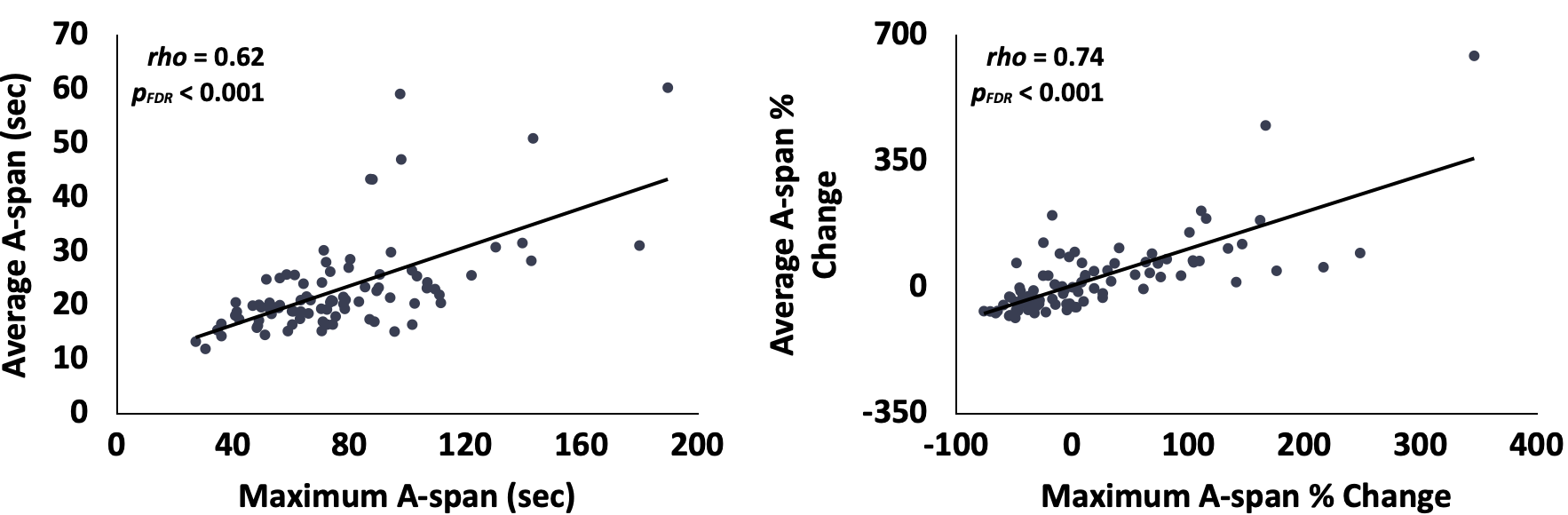


**Supplemental figure 2.** Relationships between maximum and average A-span when computed **A)** across the whole task, and **B)** when assessing the percent change from the first half to the second half.

**Age-related differences in traditional metrics**

The age-group effect for RT reported in the main text was driven by children having slower RTs than young adults (**Supplemental figure 3a**; *t*(75.81) = 9.83, *p* < 0.001, *d* = 1.77), and by older adults having slower RTs than young adults (**Supplemental figure 3a**; *t*(192) = -2.28, *p* = 0.024, *d* = -0.33). The age-effect for RTV was driven by children having a larger RTV than young adults (**Supplemental** **Figure 3b**; *t*(78.30) = 14.42, *p* < 0.001, *d* = 2.59), while RTV was not significantly different between older adults and young adults (p > 0.05).

As for the vigilance decrements in traditional metrics, the age-group effect on RT percent change was driven by children having more negative RT percent changes than young adults (**Supplemental figure 3c**; *t*(91.52) = -2.85, *p* = 0.005, *d* = 0.50), while older adults and younger adults did not significantly differ on this metric (p > 0.05). There was not an effect of age-group on RTV percent change (**Supplemental figure 3d**).

**
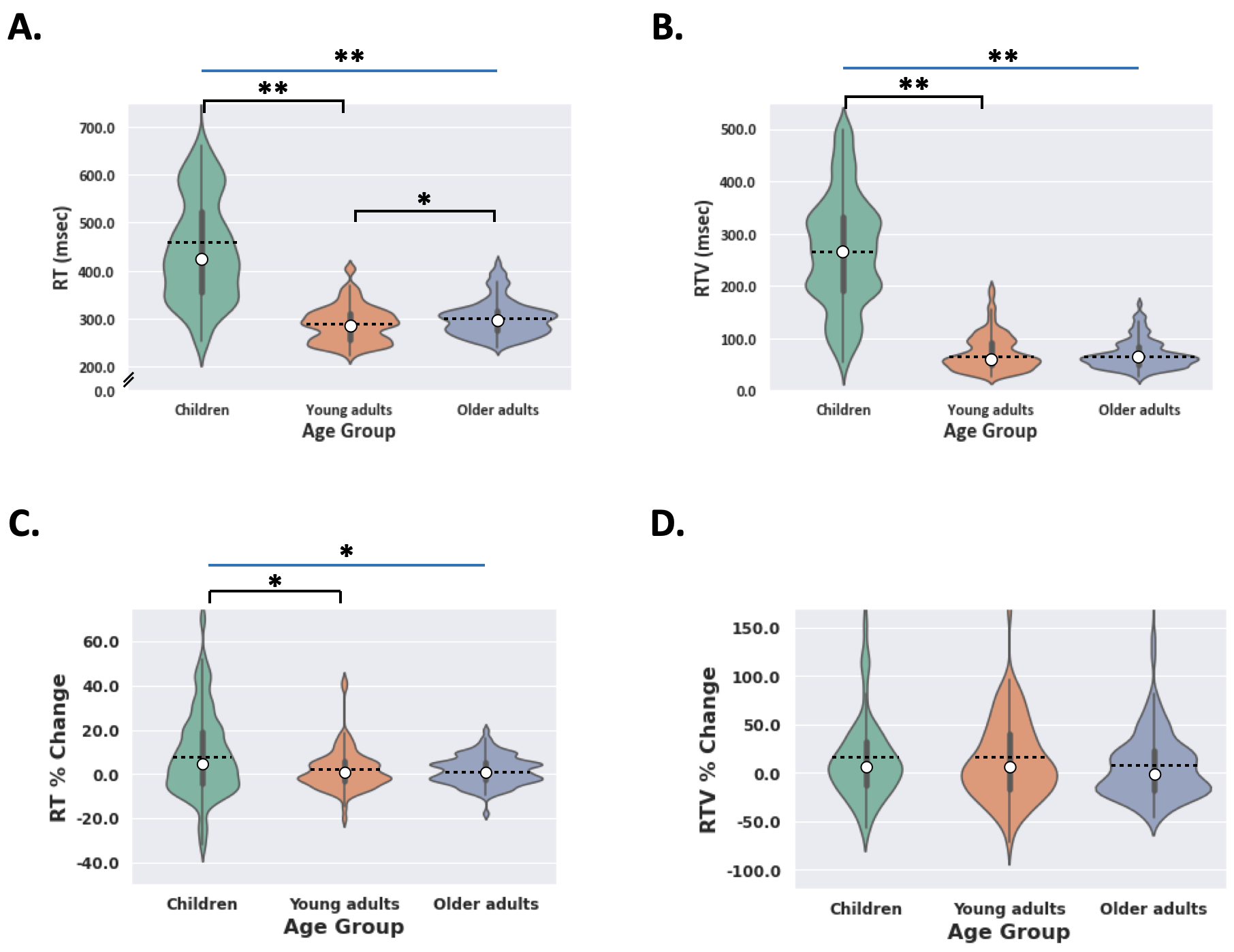
**

**Supplemental figure 3.** Age group effects on traditional metrics. **A)** Age effects on RT was driven by children and older adults having longer RTs than young adults. **B)** Age effects on RTV was driven by children having larger RTVs than young adults. **C)** Age effects in RT % change was driven by children having greater vigilance decrements (i.e., higher percent changes) than young adults. **D)** There were no age effects in RTV % change. Box and whisker plots represent the bounds of each quartile. Dashed lines represent the group average. White dots represent the group median. Blue significance bars indicate significant interactions revealed from the ANOVAs and black significance bars indicate significant t-test results. **p* < 0.05, ***p* < 0.01

**Inattention symptoms are uncorrelated with traditional metrics in children**

We sought to confirm that the traditional metrics showed expected relationships with inattention symptoms in children [8, 9]. Unexpectedly, we did not find any significant relationships between inattention symptoms, as indexed by the VADRS-IA score, and any of the traditional SA metrics (**Supplemental** **Figure 4a-d**; **a.** RT: *rho(44)* = 0.19, *p_FDR_ =* 0.603; **b.** RTV: *rho(44)* = 0.05, *p_FDR_ =* 0.766; **c.** RT percent change: *rho(44)* = 0.12, *p_FDR_ =* 0.603; **d.** RTV percent change: *rho(44)* = 0.15, *p_FDR_ =* 0.603).

**
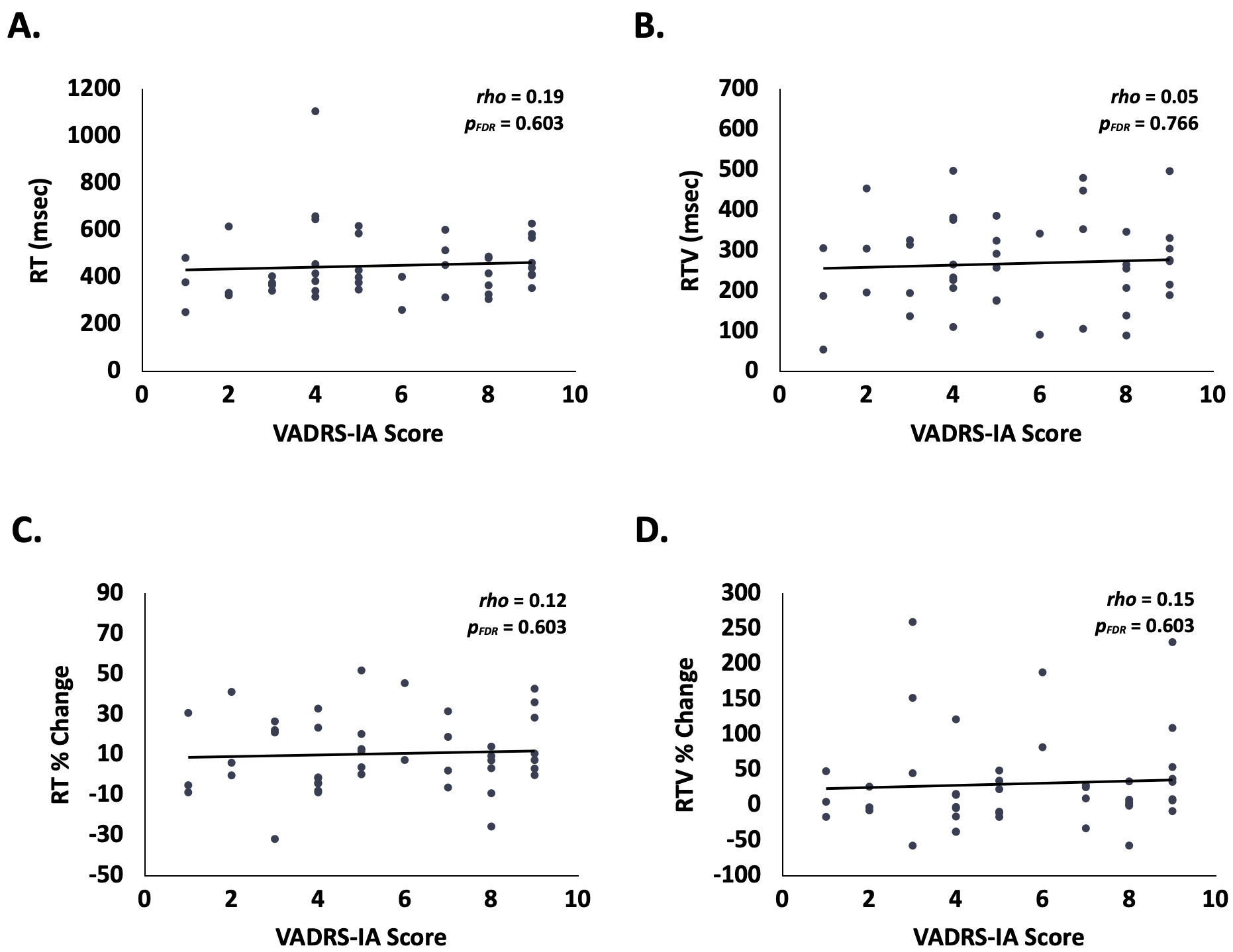
**

**Supplemental figure 4.** Relationships between inattention symptoms and traditional metrics in children. There were no significant correlations between the VADRS-IA score and **A)** RT, **B)** RTV, **C)** RT percent change, and **D)** RTV percent change. **References**

1. Gallen, C.L., et al., *Enhancing neural markers of attention in children with ADHD using a digital therapeutic.* PLoS One, 2021. **16**(12): p. e0261981.

2. Mishra, J., et al., *Closed-Loop Neurofeedback of alpha Synchrony during Goal-Directed Attention.* J Neurosci, 2021. **41**(26): p. 5699-5710.

3. Ziegler, D.A., et al., *Closed-loop digital meditation improves sustained attention in young adults.* Nat Hum Behav, 2019. **3**(7): p. 746-757.

4. Anguera, J.A., et al., *Integrated cognitive and physical fitness training enhances attention abilities in older adults.* NPJ Aging, 2022. **8**(1): p. 12.

5. Esterman, M., et al., *In the zone or zoning out? Tracking behavioral and neural fluctuations during sustained attention.* Cereb Cortex, 2013. **23**(11): p. 2712-23.

6. Leark, R., Dupuy, TR., Greenberg, LM., Kindschi, CL., Hughes, SJ., *T.O.V.A.® Professional Manual.* The TOVA Company, 2007.

7. Rosenberg, M., et al., *Sustaining visual attention in the face of distraction: a novel gradual-onset continuous performance task.* Atten Percept Psychophys, 2013. **75**(3): p. 426-39.

8. Huang-Pollock, C.L., et al., *Evaluating vigilance deficits in ADHD: a meta-analysis of CPT performance.* J Abnorm Psychol, 2012. **121**(2): p. 360-71.

9. Huang-Pollock, C.L., J.T. Nigg, and J.M. Halperin, *Single dissociation findings of ADHD deficits in vigilance but not anterior or posterior attention systems.* Neuropsychology, 2006. **20**(4): p. 420-9.
